# Nicotine Suppressed Fetal Adrenal StAR Expression via YY1 Mediated-Histone Deacetylation Modification Mechanism

**DOI:** 10.3390/ijms17091477

**Published:** 2016-09-03

**Authors:** Lian Liu, Jian-Fei Wang, Jie Fan, Yi-Song Rao, Fang Liu, You-E Yan, Hui Wang

**Affiliations:** 1 Department of Pharmacology, Basic Medical School of Wuhan University, Wuhan 430071, China; zifanqie_00@126.com (L.L.); wangjianfay@163.com (J.-F.W.); fanjcn@163.com (J.F.); raoyisong@whu.edu.cn (Y.-S.R.); liufang1020@126.com (F.L.); wanghui19@whu.edu.cn (H.W.); 2 Department of Pharmacology, Medical School of Yangtze University, Jingzhou 434000, China

**Keywords:** nicotine, Yin Yang 1 (YY1), steroidogenic acute regulatory (StAR), fetal adrenal, histone deacetylation

## Abstract

Steroidogenic acute regulatory (StAR) protein plays a pivotal role in steroidogenesis. Previously, we have demonstrated that prenatal nicotine exposure suppressed fetal adrenal steroidogenesis via steroidogenic factor 1 deacetylation. This study further explored the potential role of the transcriptional repressor Yin Yang 1 (YY1) in nicotine-mediated StAR inhibition. Nicotine was subcutaneously administered (1.0 mg/kg) to pregnant rats twice per day and NCI-H295A cells were treated with nicotine. StAR and YY1 expression were analyzed by real-time PCR, immunohistochemistry, and Western blotting. Histone modifications and the interactions between the YY1 and StAR promoter were assessed using chromatin immunoprecipitation (ChIP). Prenatal nicotine exposure increased YY1 expression and suppressed StAR expression. ChIP assay showed that there was a decreasing trend for histone acetylation at the StAR promoter in fetal adrenal glands, whereas H3 acetyl-K14 at the YY1 promoter presented an increasing trend following nicotine exposure. Furthermore, in nicotine-treated NCI-H295A cells, nicotine enhanced YY1 expression and inhibited StAR expression. ChIP assay showed that histone acetylation decreased at the StAR promoter in NCI-H295A cells and that the interaction between the YY1 and StAR promoter increased. These data indicated that YY1-medicated histone deacetylation modification in StAR promoters might play an important role in the inhibitory effect of nicotine on StAR expression.

## 1. Introduction

The adrenal gland is a terminal effector of the hypothalamic-pituitary-adrenal axis and plays a pivotal role, predominantly via steroidogenesis, in the regulation of intrauterine homeostasis and in fetal development and maturation [1,2,3]. Long-term consequences of low birth weight on adrenal cortisol secretion contribute to increased risks for metabolic syndrome in later life, suggesting a central role for adrenocortical steroidogenesis in intrauterine fetal programming [4]. Similar to adults, human fetal adrenal glands express the steroidogenic acute regulatory (StAR) gene, which is indispensable for the biosynthesis of steroid hormones [5]. StAR mediates the rate-limiting step in steroidogenesis: the transfer of cholesterol from the outer mitochondrial membrane to the inner mitochondrial membrane [6]. Our previous studies have shown that, in addition to nicotine, prenatal exposure to ethanol could inhibit adrenal StAR expression and induce intrauterine growth retardation (IUGR) in fetal rats [7,8]. Furthermore, our results have also shown that nicotine-induced CpG methylation of the Pax6 binding motif in the *StAR* promoter reduced gene expression and cortisol production [9]. We have also confirmed that the nicotine-mediated reduction of steroidogenic factor-1 (SF-1) expression resulted in inhibitory effects on the expression of various steroidogenic enzymes and steroid production via the action of histone deacetylases (HDACs) [10]. However, it has been shown that the *StAR* gene is subject to both positive and negative regulation and the mechanism of its regulation is quite complex [11]. One major control point for StAR synthesis occurs at the level of transcription [12].

Yin Yang 1 (YY1), a ubiquitous transcription factor found in normal and cancerous cells, plays an important role in cytokinesis, apoptosis, cell development, and cell differentiation [13]. It has various biological functions and has a pleiotropic effect on promoters and gene expressions [14]. YY1 can initiate, activate, or repress gene transcription by its interaction with available cofactors [15]. Previous reports have suggested YY1 is a potential suppressor of StAR transcription [16,17]. Multiple mechanisms for YY1 inhibition of transcription have been proposed, and three possible mechanisms are as follows. The first mechanism involves YY1 obstructing its recognition site by binding to an activator [18]. The second mechanism involves YY1 directly binding to a promoter to bend the DNA and interfering with activator interaction [19]. The third mechanism involves YY1-mediated recruitment of histone deacetylases, which results in transcriptional silencing [20]. It has been shown that YY1, with HDACs as corepressors, was a crucial negative transcriptional regulator of glutamate transporter (EAAT2) and mediated manganese-induced EAAT2 repression [21]. Multiple potential YY1 binding sequences have been identified in the StAR promoter [16,22]. YY1 has also been shown to increase HDAC activity [23]. Nicotine has been reported to induce chromatin decondensation and histone H3 acetylation [24]. Using a nicotine-conditioned place preference protocol, Pastor et al. demonstrated that HDAC2 was involved in promoting synaptic plasticity, which was responsible for the preference for nicotine [25]. Therefore, we speculate that YY1 mediated-recruitment of histone deacetylases might play a role in nicotine-mediated StAR inhibition.

Chromatin immunoprecipitation (ChIP) is a widely used technique which can determine the interactions of proteins with specific genomic DNA regions both in vivo and in vitro [26]. Over the years, this versatile technique has been used to identify the transcription factor binding sites or post-translational modifications on histones in diverse cellular processes [27,28]. Therefore, based on the ChIP technique, we demonstrated that YY1 mediated the inhibitory function of nicotine on StAR expression in adrenal glands of fetal rats and NCI-H295A cells, and characterized the mechanisms of this inhibition in the present study. These findings provide insight into the negative regulatory mechanism of StAR expression and help explain nicotine-induced susceptibility toward adult metabolic syndrome in intrauterine growth retardation (IUGR) offspring.

## 2. Results

### 2.1. Expression Patterns of Steroidogenic Acute Regulatory (StAR) and Yin Yang 1 (YY1) in Nicotine-Treated Fetal Rat Adrenal Glands

**Figure 1:**
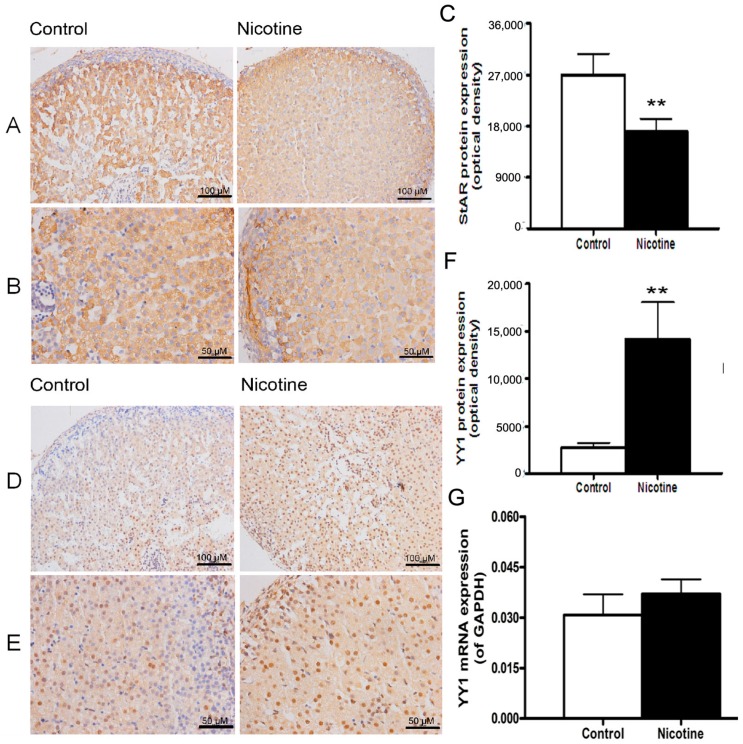
Effects of prenatal nicotine exposure on steroidogenic acute regulatory (StAR) and Yin Yang 1 (YY1) expression in fetal rat adrenal glands. (**A**,**D**) Representative immunohistochemical staining (×200), brown colors indicate the positive area; (**B**,**E**) representative immunohistochemical staining (×400); (**C**,**F**) Mean optical density analysis for StAR and YY1 protein expression; (**G**) quantitative real-time PCR (qRT-PCR) analysis for YY1 mRNA expression. Data are presented as the mean ± standard error of the mean (SEM), *n* = 5. ** *p* < 0.01 vs. control.

In our previous study, prenatal nicotine exposure inhibited the expression of StAR mRNA in fetal adrenal glands [10]. In the present study, we found that prenatal nicotine exposure also suppressed StAR protein expression (*p* < 0.01, Figure 1A–C). Compared with the control group, a 0.63-fold decrease in StAR protein levels was found in the prenatal nicotine-exposed group (*p* < 0.01, Figure 1C). As shown in Figure 1D–G, compared with the control groups, a 1.2-fold increase in YY1 mRNA levels (Figure 1F) and 5.2-fold increase in YY1 protein levels (*p* < 0.01, Figure 1G) were observed in the prenatal nicotine-exposed group. These results suggested that changes in YY1 expression were inversely related to changes in StAR expression.

### 2.2. Histone Modification of StAR and YY1 in Fetal Rat Adrenal Glands

**Figure 2:**
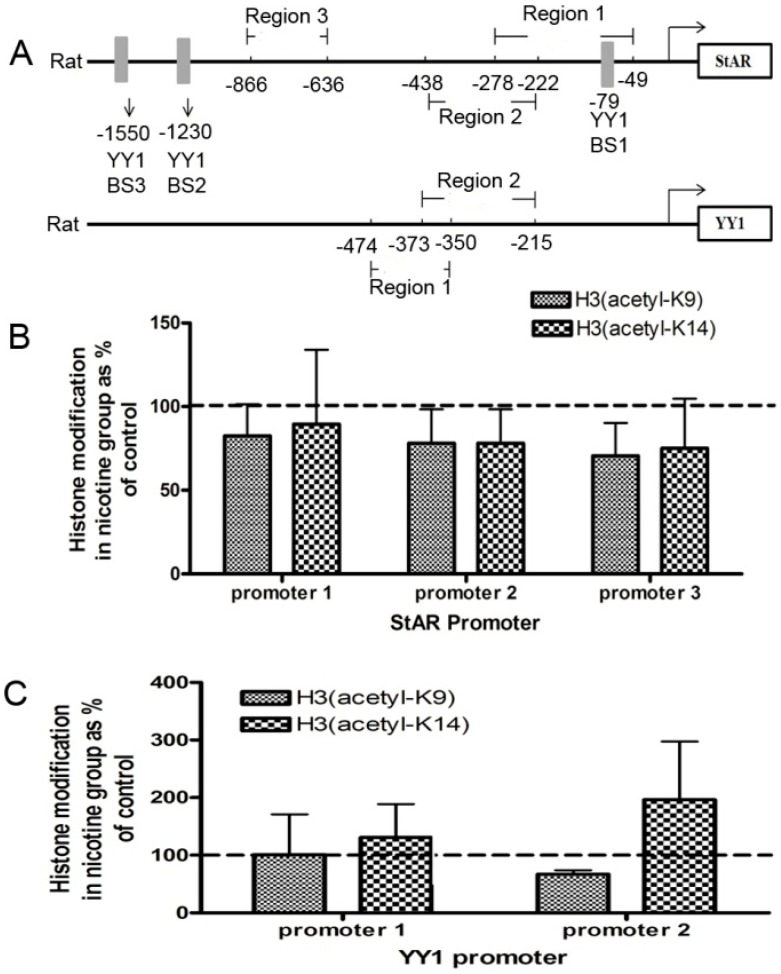
Effects of prenatal nicotine exposure on histone modification of the steroidogenesis acute regulatory protein (StAR) and Yin Yang 1 (YY1) promoters in fetal rat adrenal glands. (**A**) Schematic of the rat StAR and YY1 promoter structure; three potential YY1 binding regions (YY1 BS 1–3) are indicated; (**B**) chromatin immunoprecipitation (ChIP) analysis for histone acetylation levels in StAR promoters; (**C**) ChIP analysis for histone acetylation levels in YY1 promoters. After immunoprecipitating the complex with antibodies against acetyl histone H3K9, H3K14, or IgG, we detected the mRNA expression of StAR and YY1 using qRT-PCR. Data are presented as the mean ± SEM, all fetal adrenal glands of different pups from the same pregnant rats were merged into one sample, *n* = 3; dashed line indicated the control group was 100%.

Histone modification patterns on the StAR and YY1 promoter regions in nicotine-treated fetal adrenal glands were determined using ChIP assays. A schematic of the rat StAR and YY1 promoter structure is shown in Figure 2A. Three PCR primer pairs were generated to cover three regions in the rat StAR promoter. Two PCR primer pairs were generated to cover two regions in the rat YY1 promoter. Two antibodies against acetyl histone H3 (H3 acetyl-K9, H3 acetyl-K14) were used in the ChIP assays. As shown in Figure 2B, there was a decreasing trend for H3 acetyl-K9 in StAR promoter region 1 (−278 to −49, −18%), StAR promoter region 2 (−438 to −222, −22%) and StAR promoter region 3 (−866 to −636, −29.5%). Similarly, there was a decreasing trend for H3 acetyl-K14 in StAR promoter region 1 (−278 to −49, −11%), StAR promoter region 2 (−438 to −222, −17%) and StAR promoter region 3 (−866 to −636, −25%). Figure 2C showed that there was an increasing trend for H3 acetyl-K9 in YY1 promoter region 2 (−373 to −215, −29.5%). Additionally, there was a decreasing trend for H3 acetyl-K14 in YY1 promoter region 1 (−474 to −350, −23%) and an increasing trend for H3 acetyl-K14 in YY1 promoter region 2 (−373 to −215, +94%). These results indicated that histone acetylation in StAR and YY1 promoters in fetal rat adrenal glands were modified by prenatal nicotine exposure.

### 2.3. Expression Patterns of StAR and YY1 in Nicotine-Treated NCI-H295A Cells

**Figure 3:**
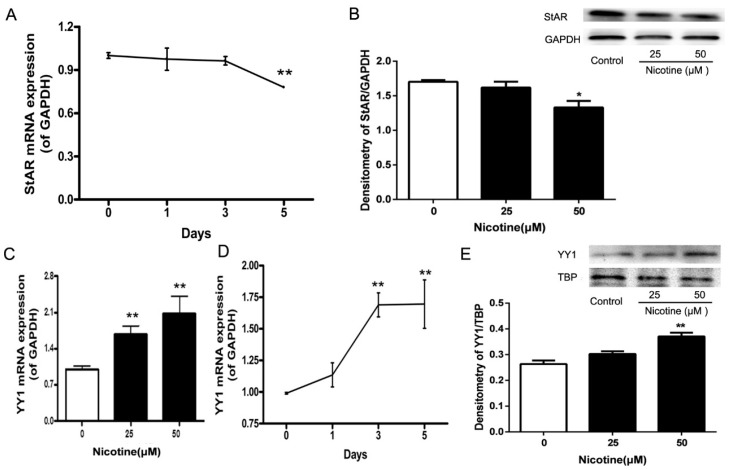
Time- and concentration-dependent effects of nicotine treatment on the expression of steroidogenic acute regulatory protein (StAR) and Yin Yang 1 (YY1) promoters in NCI-H295A cells. (**A**) qRT-PCR analysis for StAR mRNA expression (in a time-dependent manner); (**B**) Western blotting analysis for StAR protein expression; (**C**) qRT-PCR for YY1 mRNA expression (in a concentration-dependent manner); (**D**) qRT-PCR analysis for YY1 mRNA expression (in a time-dependent manner); (**E**) Western blotting analysis for YY1 protein expression. GAPDH: glyceraldehyde-phosphate dehydrogenase; TBP: TATA-binding protein. Data are presented as the mean ± SEM, *n* = 5. * *p* < 0.05, ** *p* < 0.01 vs. control.

In our previous study, we found that a nicotine treatment of 50 μM for 5 days inhibited StAR mRNA expression [10]. In the current study, we further confirmed that a nicotine treatment of 50 μM for 5 days inhibited StAR mRNA expression to 64.56% (*p* < 0.01, Figure 3A). Nicotine treatment of 50 μM for 5 days also inhibited StAR protein expression when compared with the control (*p* < 0.05, Figure 3B).

Compared with the controls, nicotine enhanced YY1 mRNA expression by 168.9% (*p* < 0.01) and 169.5% (*p* < 0.01) at a concentration of 50 μM for 3 and 5 days, respectively (Figure 3C), and by 167.2% (*p* < 0.01) and 194.2% (*p* < 0.01) at respective concentrations of 25 and 50 μM for 5 days (Figure 3D). Treatment with 50 μM nicotine for 5 days increased YY1 protein expression when compared with the control treatment (*p* < 0.01, Figure 3E).

### 2.4. Histone Modification of StAR in Nicotine-Treated NCI-H295A Cells

**Figure 4:**
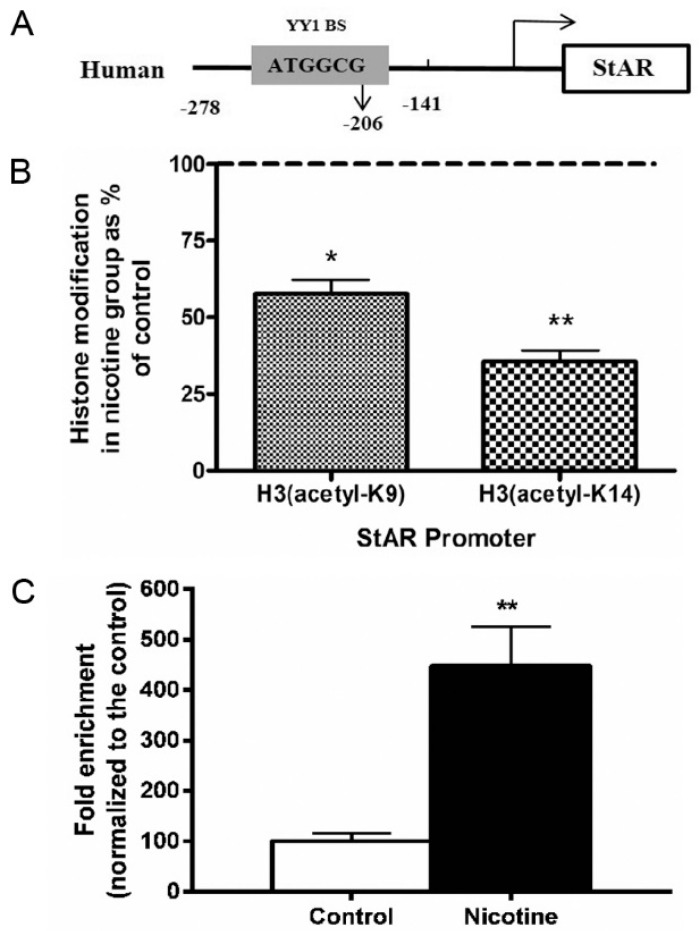
Effects of prenatal nicotine exposure on histone modification of the steroidogenesis acute regulatory protein (StAR) promoter and Yin Yang 1 (YY1)/steroidogenic acute regulatory (StAR) interaction in NCI-H295A cells. (**A**) Schematic of the human StAR promoter structure; (**B**) ChIP analysis for histone acetylation levels in StAR promoters; (**C**) ChIP analysis for the interaction between YY1 and StAR promoter. The NCI-H295A cells were treated with nicotine (50 μM) for five days. After immunoprecipitating the complex with antibodies against acetyl histone H3K9, H3K14, IgG, or YY1, we detected the mRNA expression of StAR using qRT-PCR. Data are presented as the mean ± SEM, *n* = 3. * *p* < 0.05, ** *p* < 0.01 vs. control. Dashed line indicated the control group was 100%.

A schematic of the human StAR promoter structure is shown in Figure 4A. One PCR primer pair was generated to cover one region in the human StAR promoter. Two antibodies against acetyl histone H3 (H3 acetyl-K9, H3 acetyl-K14) were used in the ChIP assays. ChIP assay results showed that nicotine treatment decreased the levels of H3 acetyl-K9 (−22%, *p* < 0.05) and H3 acetyl-K14 (−64%, *p* < 0.01) in the StAR promoter region (−278 to −141), respectively (Figure 4B). In our previous study, treatment with TSA, a deacetylation inhibitor, reversed nicotine-mediated StAR inhibition [10]. These results indicated that the nicotine treatment induced histone deacetylation at the StAR promoter region in NCI-H295A cells.

### 2.5. YY1 and StAR Promoter Interaction in Nicotine-Treated NCI-H295A Cells

Based on the gene sequence analysis, we found a prediction of transcription factor YY1 binding sites (−206 to −211, ATGGCG) in the human StAR promoters (Figure 4A). To determine whether nicotine inhibits StAR through the effects of YY1, we investigated the interaction between YY1 and the StAR promoter by performing ChIP in NCI-H295A cells. After immunoprecipitating the complex with antibodies against YY1, we observed the mRNA expression of StAR using qRT-PCR in the control and nicotine treatment group, which identified the interaction between YY1 and StAR promoter (Figure 4C). Furthermore, when compared with the controls, the fold enrichment was significantly increased by 1.86 ± 0.40-fold in the nicotine treatment group (Figure 4C, *p* < 0.01), which indicated the increasing interaction between YY1 and StAR promoter.

## 3. Discussion

Several factors may contribute to the association between maternal smoking and negative outcomes in offspring. Among them, nicotine is considered to be one of the major aversive components, and the dose of nicotine is an important consideration [29]. According to the previous study, the dose (2 mg/kg/day) of nicotine used in the in vivo animal experiment in our study could produce similar plasma nicotine levels with those observed in moderate to heavy smokers [30,31,32]. Typically, plasma nicotine levels in females who were heavy smokers were found to range between 0.3 and 0.6 µM [30]. However, research also demonstrated that a smoker would accumulate approximately 100 μM of nicotine in the saliva when smoking 25 cigarettes per day [33]. Based on the plasma levels examined in active and passive smokers, Zhang et al. chose concentrations of nicotine ranging from 0.01 to 100 μM in human subretinal pigment epithelium cell line experiments [34]. In other in vitro experiments, the concentration of nicotine used was also as high as 100 μM [35]. In our in vitro cell culture experiments, the concentration of nicotine (25 and 50 μM) is higher than that reported in heavy smokers (0.3 to 0.6 μM) [30]. However, the concentration of nicotine in amniotic fluid, fetal blood, or fetal tissue during pregnancy may increase due to the high liposolubility of nicotine, which allows it to pass through the placenta and increases its accumulation in the fetus. On the other hand, the expression level of cytochrome P450 2A6, a kind of nicotine metabolic enzyme, is very low in the fetus [36].

In this study, we found that prenatal nicotine exposure increased YY1 expression and suppressed StAR expression. ChIP assay showed that histone acetylation in the StAR and YY1 promoter in fetal rat adrenal glands were modified by prenatal nicotine exposure. There was a decreasing trend of histone acetylation at the StAR promoter region in fetal adrenal glands, whereas histone acetylation at the YY1 promoter presented an increasing trend following nicotine exposure. Furthermore, in nicotine-treated NCI-H295A cells, nicotine enhanced YY1 expression and inhibited StAR expression. ChIP assay showed that histone acetylation at the StAR promoter in NCI-H295A cells decreased and that the interaction between YY1 and StAR promoter increased. These data indicated that histone acetylation modification in YY1 and StAR promoter may play an important role in the suppression of fetal adrenal StAR expression following nicotine treatment.

Several studies have reported that YY1 could regulate the expression of various genes and that it was crucial for embryonic development [13]. YY1 functions as a transcriptional repressor for a variety of genes, including StAR [17], Flap endonuclease 1 (FEN1) [37], and prostate stem cell antigen [38]. Recent studies have shown that the ectopic expression of YY1 prevented CCAAT/enhancer-binding protein β from binding to the peroxisome proliferative-activated receptor γ (PPARγ) promoter, resulting in down-regulation of PPARγ transcriptional activity in 3T3-L1 cells [39]. The activity of YY1 as a transcription factor could be regulated by gene expression, protein cellular localization, and/or the discriminatory binding of cofactors. YY1 was up-regulated by interleukin-13 in lung fibroblasts in a dose- and time-dependent manner [40]. Changes in YY1 expression have been observed during adipocyte differentiation [39] and in oncogenesis [41]. Wang et al. found that tyrosine phosphorylation led to the down-regulation of YY1 activity and that YY1 is a downstream target of epidermal growth factor receptor signaling in vivo [42]. In our study, we found that nicotine exposure increased YY1 expression in vivo and in vitro. In our previous study, we found that nicotine treatment significantly increased the expression of different HDAC subtypes [10]. Here, our data also showed that there was an increasing trend for H3 acetyl-K14 at the YY1 promoter region in fetal rat adrenal glands following prenatal nicotine exposure. Taken together, these results suggested that HDAC-mediated histone acetylation regulation may have an effect on YY1 expression during nicotine exposure.

The role of YY1 in the regulation of StAR transcription is supported by the finding that endogenous YY1 bound to the −78 cAMP response element and was recruited to the StAR promoter following angiotensin II treatment of H295R cells [43]. YY1 has also been shown to mediate prostaglandin F 2α (PGF2α)-dependent repression of the *StAR* gene expression in rat luteal cells by a direct DNA-binding mechanism [22]. In the present study, we have shown that nicotine enhanced YY1 mRNA levels and protein expression and inhibited StAR mRNA levels and protein expression, and that changes in YY1 expression were inversely related to StAR expression. In addition, based on the gene sequence analysis, we found a prediction of transcription factor YY1 binding sites (−206 to −211, ATGGCG) in the human StAR promoters. We also found that the interaction between the YY1 and StAR promoter was enhanced following nicotine treatment in our study. Taken together, these data indicated that there was an interaction between the YY1 and StAR promoter regions. Thus, it is tempting to speculate that YY1 acts as a negative transcription factor at the StAR promoter, suppressing StAR transcription in NCI-H295A cells and in fetal rat adrenal glands.

**Figure 5:**
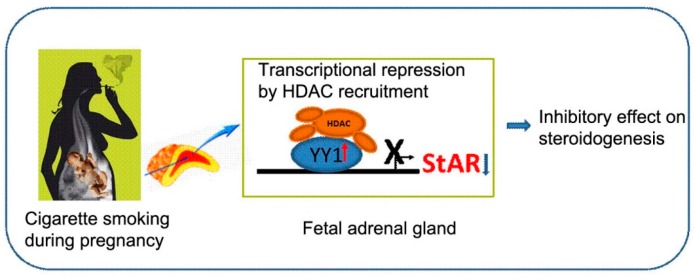
Proposed mechanism for the suppression of fetal adrenal steroidogenesis acute regulatory protein (StAR) expression following nicotine treatment. Nicotine treatment induced YY1 expression. Then YY1, acting as a negative transcription factor at the StAR promoter, recruited HDACs to the promoter of StAR and suppressed its transcription and steroidogenesis. YY1: Yin Yang 1; HDAC: histone deacetylases. Red arrow indicates gene expression increases and blue arrow indicates gene expression decreases.

Epigenetic mechanisms have been reported to regulate the gene expression of StAR in the ovary and in several other types of cells [44,45]. Hiroi et al. suggested that a combinatorial code of transcription factors, including reciprocal changes in histone modifications associated with active transcription and gene silencing, control *StAR* gene expression [46]. Lee et al. provided evidence that histone modifications were involved in rapid changes in *StAR* gene expression during ovulation [47]. In the present study, we also found that H3 acetyl-K9 and H3 acetyl-K14 were reduced in the StAR promoter region in NCI-H295A cells and fetal rat adrenal glands following nicotine treatment, and that nicotine exposure suppressed StAR mRNA levels and protein expression. In our previous study, treatment with TSA, a deacetylation inhibitor, reversed nicotine-mediated StAR inhibition [10]. These results indicated that nicotine treatment induced histone deacetylation in the StAR promoter, which might be involved in the inhibition of StAR expression by nicotine. Promoter region-specific changes in histone modification of the StAR gene were also observed in a mouse model [46]. Sufficient evidence has suggested that the recruitment of HDAC contributed to the suppressive effects of YY1 on various promoters [23]. It is reported that HDACs closely interacted with astrocytic YY1 and nuclear factor-κB, leading to the modulation of excitatory amino acid transporter 2 promoter activity [21]. Lu et al. have suggested that Sin3a, HDAC1, and YY1 are co-factors for Gon4l and that Gon4l might function as a platform for the assembly of complexes that regulate gene expression [48]. YY1 repressed Hoxa11-mediated downstream target gene transcription by recruiting and competing with HDACs [49]. YY1 functioned as a repressor of transcription in differentiated H9C2 cells via its interaction with HDAC5 [50]. The cooperative silencing effect of Myc and YY1 on the integrin α3 gene involved deacetylation activity in the promoter [51]. In our previous study, we found that nicotine treatment significantly increased the expression of HDAC2 in fetal adrenal glands and enhanced HDAC1, HDAC2, and HDAC7A mRNA expression in NCI-H295A cells [10]. Liu et al. reported that PGF2α enhanced a direct YY1/StAR promoter interaction and the recruitment of HDAC1 to the promoter, thereby preventing transcriptional activation of the *StAR* gene [22]. Thus, we speculated that YY1 mediated-recruitment of histone deacetylases might play a role in nicotine-mediated StAR inhibition (Figure 5).

Taken together, these findings demonstrated that nicotine exposure induced YY1 expression and suppressed StAR expression in fetal rat adrenal glands in vivo and in NCI-H295A cells in vitro. We have also indicated that YY1-medicated histone deacetylation modification in the StAR promoter might play an important role in the suppression of fetal adrenal StAR expression following nicotine treatment. However, further research and confirmation is needed to provide direct evidence of this. Our results should provide a better understanding of the regulation of *StAR* gene expression in fetal adrenal glands.

## 4. Materials and Methods

### 4.1. Animals and Treatment

Pathogen-free Wistar rats weighing 180–220 g (female) or 260–300 g (male) were obtained from the Experimental Center of Hubei Medical Scientific Academy (No. 2006-0005, Wuhan, China). The animals were allowed to acclimate for one week before being subjected to experimental conditions. All animal studies were performed at the Center for Animal Experimentation of Wuhan University (Wuhan, China), which has been accredited by the Association for the Assessment and Accreditation of Laboratory Animal Care International (AAALAC International). The study protocol was designed in accordance with the Guidelines for Animal Research and was approved by the Ethics and Research Committee of the Medical College of Wuhan University (No. 07006, 20 March 2007).

Each male rat was mated with two female rats, and the occurrence date of vaginal plug formation or identification of sperm in a vaginal smear was considered gestational day (GD) 0. Pregnant rats were then transferred to individual cages and randomly divided into two groups: control group and nicotine group, and 5 dams were in each group. Nicotine (Sigma Chemical Co., St. Louis, MO, USA) was subcutaneously administered (1.0 mg/kg) to pregnant rats twice per day from GD 11 to 20 to establish a stable IUGR rat model [52], and the control group was administered the same volume of distilled water. On GD20, the animals were anesthetized and rapidly euthanized by bleeding from the left carotid artery. In general, the pregnant rats produced 10–12 pups. Each feto-placental unit was quickly removed from the uterus, and fetal adrenal glands were collected. One pair of fetal adrenal glands was randomly selected and fixed in phosphate-buffered 4% paraformaldehyde solution for 24 h. The remaining adrenal glands from littermates were pooled together and immediately frozen in liquid nitrogen and then stored at −80 °C for subsequent experiments.

### 4.2. Human Adrenocortical Carcinoma (NCI-H295A) Cell Culture and Drug Treatment

Standard media was used for NCI-H295A cells, consisting of RPMI-1640 supplemented with 2% FBS (Gibco, Auckland, New Zealand), 0.1% SIT, and penicillin/streptomycin. When the NCI-H295A cells reached 40% confluence, they were starved in serum-free medium overnight and then treated with nicotine (25 and 50 μM) for five days (*n* = 5). A concentration of 50 μM nicotine was used in the time course experiment (*n* = 5). NCI-H295A cells, total RNA, and protein were stored at −80 °C for future analysis.

### 4.3. Immunohistochemistry Observations

Immunohistochemical procedures were performed using a streptavidin-peroxidase (SP)-conjugated method according to the manufacturer’s instructions. Sections were incubated at 37 °C for 20 min with rabbit polyclonal antibodies against StAR (sc-25806, 1:400) or YY1 (sc-1703, 1:400) (Santa Cruz, CA, USA). Negative controls were prepared by omitting the primary antibody. Under light microscopy, the expression of YY1 or StAR was observed and microphotographs were taken. The cells were considered positive for YY1 only when there was a clear nuclear staining. The cells were considered positive for StAR only when there was a clear cytoplasm staining. Image quantification was carried out using an Image-Pro Plus 6.0 (Media Cybernetics, Silver Spring, MD, USA). By placing marks of brown colors onto the positive area, optical density values per unit of positive area in each view field were obtained automatically. For each section, 5 fields were examined. In each group, the final quantitative results were the average values of the five immunohistochemistry slides [53].

### 4.4. Quantitative Real-Time PCR (qRT-PCR)

Total RNA was isolated from fetal adrenal glands and NCI-H295A cells using Trizol (Invitrogen, Carlsbad, CA, USA), according to the manufacturer’s protocol. Relative gene expression levels of YY1 and StAR were determined by qRT-PCR using an ABI Step One real-time PCR thermal cycler (ABI StepOne, Foster City, CA, USA). qRT-PCR analysis was performed according to a previously described method [10]. Primers were designed using Primer Premier 5.0, and their sequences and annealing temperature are shown in Table 1. The mRNA level of the housekeeping gene glyceraldehyde phosphate dehydrogenase (GAPDH) was measured and used as a quantitative control.

### 4.5. Western Blotting Analysis

Briefly, nuclear and cytoplasm extracts were prepared using a nuclear extraction kit (Bio-Rad, Hercules, CA, USA). The samples were loaded onto 10% SDS polyacrylamide gels and electrophoresed (SDS-PAGE). The proteins were transferred onto a nitrocellulose membrane and incubated with rabbit polyclonal antibodies against StAR (sc-25806, 1:800 dilution) (Santa Cruz, CA, USA), YY1 (sc-1703, 1:800 dilution) (Santa Cruz, CA, USA), GAPDH (sc-25778, 1:2000 dilution) (Santa Cruz, CA, USA), and TBP (ab63766, 1:2000 dilution) (Abcam, Cambridge, MA, USA) overnight at 4 °C. Finally, the membrane was incubated in the dark for 1 h with a fluorescent secondary antibody and specific bands were detected via electrochemiluminescence (ECL) assay. The signals of StAR (30 kDa), YY1 (68 kDa), GAPDH (37 kDa), and TBP (38 kDa) were visualized using ECL reagents (Pierce Biotechnology, Inc. Rockford, IL, USA) and then captured using a chemiluminescence and multicolor fluorescence imaging system (Fusion Fx7, Vilber Lourmat, Marne la Vallée Cedex 1, France). Specific bands were analyzed using Gel-Pro Analyzer 4.0 (Media Cybernetics, Silver Spring, MD, USA).

### 4.6. Chromatin Immunoprecipitation (ChIP)

All fetal adrenal glands of different pups from the same pregnant rats were merged into one sample in ChIP assays (*n* = 3). The protocol for ChIP was performed as previously described [54]. Briefly, chromatin obtained from fetal adrenal glands or NCI-H295A cells was cross-linked with 1.0% formaldehyde. The chromatin solution was precleared with 50 μL of protein G-agarose beads (GE Healthcare, Guangzhou, China), followed by immunoprecipitation overnight using rabbit polyclonal antibodies against acetyl histone H3K9 (ab10812), H3K14 (ab52946), YY1 (ab12132), or IgG (ab171870) (Abcam, Cambridge, MA, USA). The precipitated samples were analyzed using qRT-PCR. Sequences of the PCR primers used are shown in Table 2.

### 4.7. Statistics

Statistical analysis was performed using Prism (GraphPad Software, La Jolla, CA, USA, version 5.0). Statistical significance was established between two groups using a *t*-test and among multiple groups using analysis of variance (ANOVA). A *p*-value of less than 0.05 was considered to be statistically significant.

## 5. Conclusions

Our findings demonstrated that nicotine exposure induced YY1 expression and suppressed StAR expression in fetal rat adrenal glands in vivo and in NCI-H295A cells in vitro. We have also indicated that YY1-medicated histone deacetylation modification in the StAR promoter might play an important role in the suppression of fetal adrenal StAR expression following nicotine treatment.

## Figures and Tables

**Table 1:** Oligonucleotide primers and PCR conditions.

Genes	Forward Primer	Reverse Primer	Product (bp)	Annealing
Rat *StAR*	GGGAGATGCCTGAGCAAAGC	GCTGGCGAACTCTATCTGGGT	188	65 °C, 20 s
Rat *YY1*	CAGAAGCAGGTGCAGATCAAGAC	CCCTGAACATCTTTGTGCAGCC	307	68 °C, 30 s
Rat *GAPDH*	GCAAGTTCAACGGCACAG	GCCAGTAGACTCCACGACA	107	63 °C, 30 s
Human *StAR*	GATTTTGCCAACCACCTGC	GGATTCTCCTGATGAGCGTGT	127	60 °C, 25 s
Human *YY1*	CAGAAGCAGGTGCAGATCAAGAC	CCCTGAACATCTTTGTGCAGCC	307	68 °C, 30 s
Human *GAPDH*	GAAATCCCATCACCATCTTCCAG	ATGAGTCCTTCCACGATACCAAAG	313	59 °C, 15 s

**Table 2:** Oligonucleotide primers and conditions.

Genes	Forward Primer	Reverse Primer	Product (bp)	Annealing
Rat *StAR-1* (−278~−49)	AATGCTGAACCTGGAGCTTG	GGAAGGCTGTGCATCATCAC	229 bp	63 °C, 30 s
Rat *StAR-2* (−438~−222)	TGTCTGTTCCTGGGAGAGTTG	TCCAAAGTTTTAAATGGAGAC	216 bp	58 °C, 30 s
Rat *StAR-3* (−866~−636)	CACAGGTCAAGTCCTGTAGC	CTTGGTTCCTTCCACATGTG	230 bp	63 °C, 30 s
Rat *YY1-1* (−474~−350)	CCAGAACAGCGAGTGGGA	AACTCCTCAACCCGAGCC	124 bp	61 °C, 30 s
Rat *YY1-2* (−373~−215)	ATCTGGGCTCGGGTTGAG	CCGCTGCTTGTCCTGCTC	158 bp	64 °C, 30 s
Human *StAR* (−278~−141)	AAACGGCCAAAGCAGCAGTGTGAGG	GACCTGGTGTTCTGCGTCTT	59 bp	63 °C, 30 s
